# Supplementary material for: In Vivo and In Vitro Biological Evaluation and Molecular Docking Studies of Compounds Isolated from Micromeria biflora (Buch. Ham. ex D.Don) Benth
Source: Molecules. 2022 May 24;27(11):3377. doi: 10.3390/molecules27113377 (PMC9181852; doi:10.3390/molecules27113377)
Supplement: Supplementary file 1 [file molecules-27-03377-s001.zip › molecules-1688535-supplementary.pdf]

Supplementary Material

In Vivo and In Vitro Biological Evaluation and Molecular Docking Studies of Compounds Isolated from *Micromeria biflora* (Buch. Ham. ex D.Don) Benth

Abdullah S. M. Aljohani [1,*], Fahad A. Alhumaydhi [2], Abdur Rauf [3,*], Essam M. Hamad [4] and Umer Rashid [5]

[1] Department of Veterinary Medicine, College of Agriculture and Veterinary Medicine, Qassim University, Buraydah 52571, Saudi Arabia
[2] Department of Medical Laboratories, College of Applied Medical Sciences, Qassim University, Buraydah 52571, Saudi Arabia; f.alhumaydhi@qu.edu.sa
[3] Department of Chemistry, University of Swabi, Anbar23561, Khyber Pakhtunkhwa, Pakistan
[4] Department of Food Science and Human Nutrition, College of Agriculture and Veterinary Medicine, Qassim University, Buraydah 52571, Saudi Arabia; e.hamad@qu.edu.sa
[5] Department of Chemistry, COMSATS University Islamabad, Abbottabad Campus, 22060, Pakistan; umerrashid@cuiatd.edu.pk
* Correspondence: jhny@qu.edu.sa (A.S.M.A.); mashaljcs@yahoo.com (A.R.)

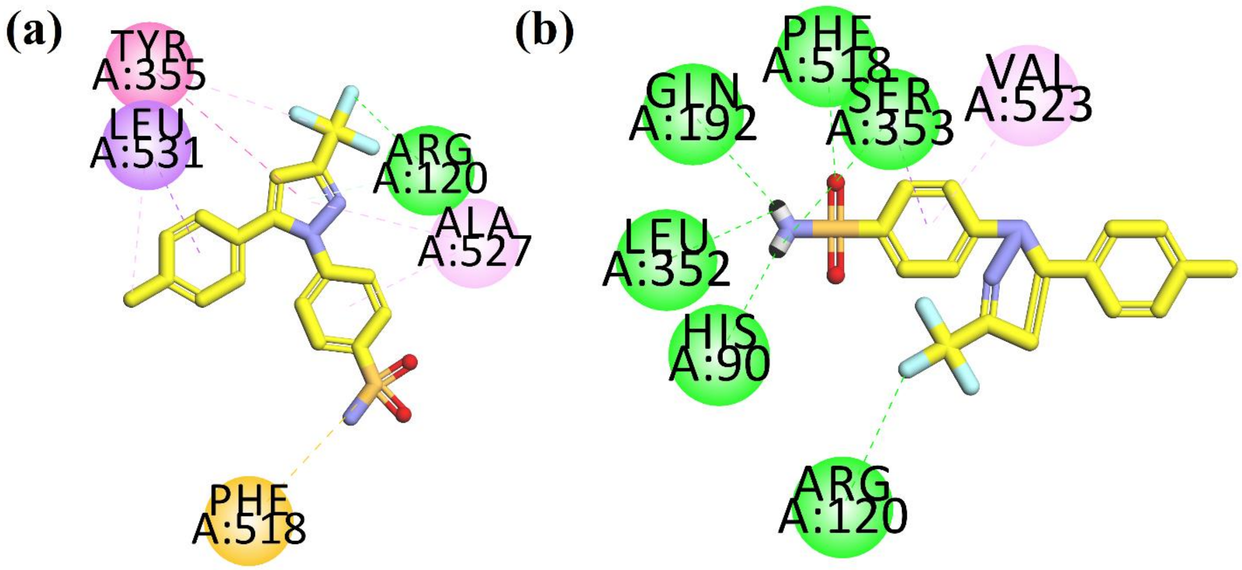

Figure S1. (**a**) The 2D interaction diagrams of isolated salicylalazine into the binding site of COX-1 (3N8Y) (**b**) COX-2 (1CX2).

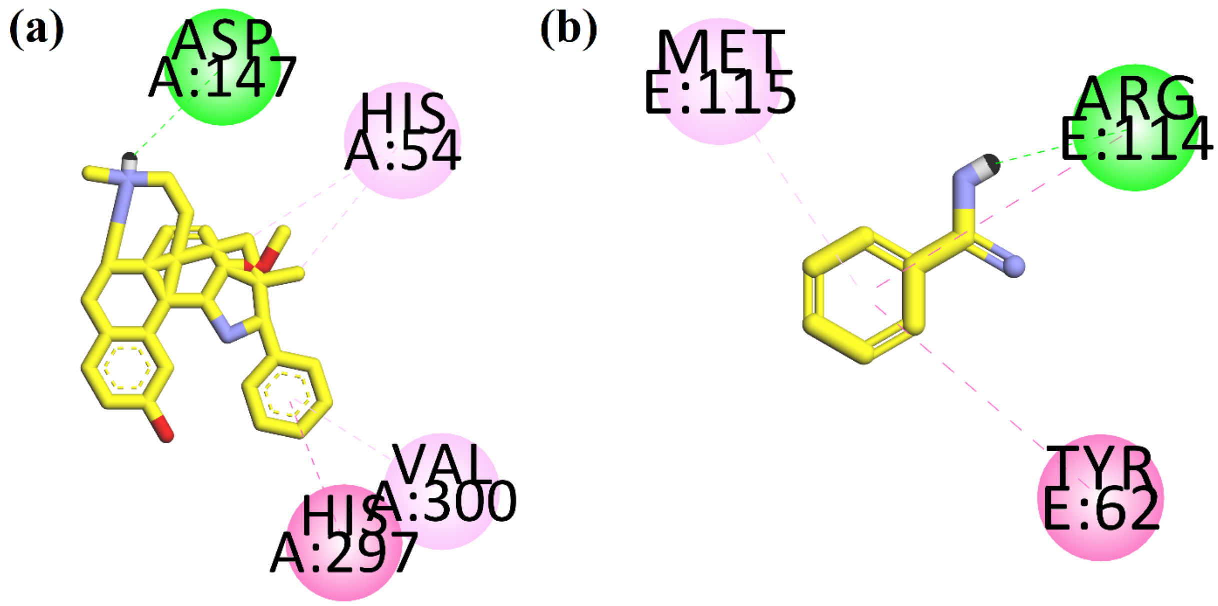

Figure S2. (**a**) The 2D interaction diagrams of isolated salicylalazine into the binding site of GABA (4COF) (**b**) μ-opioid (5C1M).
